# Predisposing factors for recurrence of chronic posttraumatic osteomyelitis: a retrospective observational cohort study from a tertiary referral center in Brazil

**DOI:** 10.1186/s13037-017-0133-1

**Published:** 2017-06-02

**Authors:** Luciana Souza Jorge, Alceu G. Chueire, Patricia Silva Fucuta, Mauricio N. Machado, Maria Gabriele L. Oliveira, Marcelo A. Nakazone, Mauro José Salles

**Affiliations:** 1 Hospital de Base, Infection Control Unit, São José do Rio Preto Medical School, São Paulo, Brazil; 2 Orthopedics and Traumatology Unit, São José do Rio Preto Medical School, São Paulo, Brazil; 3 Division of Infectious Diseases, Department of Internal Medicine, Santa Casa de São Paulo School of Medical Sciences, São Paulo, Brazil; 4 Hospital da Irmandade da Santa Casa de Misericórdia de São Paulo, Rua Dr Cesáreo Mota Jr 112, CEP: 01221-020 São Paulo, SP Brazil

**Keywords:** Posttraumatic osteomyelitis, Recurrence, Risk factors, Blood transfusion, *Pseudomonas aeruginosa*

## Abstract

**Background:**

The incidence of posttraumatic osteomyelitis (PTO) is increasing despite new treatment strategies. Assessment of patients’ outcomes following PTO is challenging due to multiple variables. The study goals are to determine the frequency of recurrence following PTO treatment and identify factors predisposing patients to treatment failure.

**Methods:**

Between August 01, 2007 to August 30, 2012, a single-center retrospective cohort study was performed among 193 patients diagnosed with PTO following orthopedic surgery for fracture care. Bone and soft tissues were collected for cultures and PTO was defined according to CDC/NHSN criteria. Patient, injury, surgery-associated variables, and microbiological records were reviewed for risk factors associated to recurrence of PTO. Univariate and multivariable analyses using logistic regression were performed, with *p <*0.05 considered significant.

**Results:**

Thirty-eight patients (20%) of 192 diagnosed and treated for PTO failed their treatment. Factors associated with recurrence were age between 61 and 80 years [hazard ratio (HR) = 6.086, 95% confidence interval (CI) = 2.459;15.061, *p* = <0.001], age above 80 years [HR = 9.975 (95% CI = 3.591;27.714), *p* = <0.001], intraoperative blood transfusion [HR = 2.239 (95% CI = 1.138;4.406), *p* = 0.020], and positive culture for *Pseudomonas aeruginosa* [HR = 2.700 (95% CI = 1.370;5.319), *p* = 0.004].

**Conclusions:**

Risk factors associated with recurrence of PTO are difficult to measure. The present study revealed that elderly patients, intraoperative blood transfusions, and infection due to *P. aeruginosa* were independently associated with recurrence of PTO. These factors should warn clinicians of a higher failure rate following treatment of PTO. Trial registration: ISRCTN71648577. Registered 18 May 2017. Retrospectively registered.

## Background

Despite improvements in preventive, diagnostic and treatment strategies there has been a steady increase in the incidence of osteomyelitis in the adult population [1, 2]. Over the last few decades the predisposing factors and causative microorganisms of osteomyelitis have changed dramatically. In the past, bone infections were mostly due to acute haematogenous spread of Gram-positive cocci, such as *Staphylococcus aureus*. In contrast, today, osteomyelitis is commonly post-traumatic, implant-related, and affects patients with chronic diabetes mellitus. By definition, posttraumatic osteomyelitis (PTO) is a microbial (bacteria or fungi) infection of bone and may lead to bone destruction, resulting from any type of trauma or nosocomial infection from the surgical treatment of trauma that allows organisms to enter bone and proliferate in traumatized tissue (5, 6). Osteomyelitis associated with fractures can cause delayed union or non-union and complicate the treatment (2). The causative organisms are typically methicillin-resistant *Staphylococcus aureus* (MRSA), biofilm-forming coagulase-negative *Staphylococcus*, or multidrug resistant (MDR) Gram-negative bacilli including *Pseudomonas aeruginosa*, *Acinetobacter baumannii*, among others [3–6].

A population-based historical cohort study performed in the United States revealed that chronic osteomyelitis (COM) almost tripled over time in the elderly, especially among diabetic patients, while it remained relatively stable among children and young adults [7]. On the other hand, a Chinese cohort study addressing the epidemiology of COM confirmed its rising frequency, especially among previously healthy young adults involved in road traffic accidents and interpersonal violence [8]. The combination of prompt diagnosis, aggressive surgical debridement, and targeted administration of antibiotics may help reduce the growing frequency of PTO, especially among populations in the developing world [9, 10]. In contrast to data showing the rising incidence of PTO, research focusing on outcomes and predictors of recurrent bone infection following PTO treatment is scarce [11]. Recurrences following treatment of lower extremity bone infection are between 20% and 30% [12]. In a retrospective study of patients with contiguous osteomyelitis, bone infected by *Pseudomonas aeruginosa* and inappropriate antibiotic therapy for *S. aureus* were independent risk factors for recurrence [13]. Furthermore, in a recent Colombian cohort study of recurrent chronic osteomyelitis, lower rates of treatment failure were observed in patients on appropriate antibiotic therapy who had been treated by infectious disease specialists in a multidisciplinary team alongside orthopedic surgeons [14]. Detailed studies that address patient, injury, microbiological findings, and surgery-related predisposing factors to recurrence of PTO are of utmost importance as they may help implement efficient and cost saving control measures to reduce the frequency of recurrent PTO on a global scale. Our study aims to: determine the frequency of recurrence following treatment of PTO, and identify subjects, injury, pathogen, and surgery-associated risk factors predisposing patients to recurrence of PTO.

## Methods

### Study design and setting

Our study is a single-center retrospective cohort study performed over a five-year period (August 01, 2007 to August 30, 2012) in a regional tertiary referral center. The dataset was retrospectively collected from an observational cohort of patients treated for PTO. The focus of data collection was to compare characteristics of patients according to outcome (treatment success or failure).

### Participants/study subjects

Of 8,098 patients undergoing orthopedic trauma surgery, 7,510 (92.7%), and 588 (7.3%) were treated for closed and open fractures respectively. Two hundred and five patients developed PTO. Inclusion criteria were patients older than 12 years of age and with at least one year of follow-up after the surgical procedures. Patients diagnosed with a previous history of infection at the same site (7 patients) or incomplete medical records (5 patients) were excluded from our study. A total of 193 patients diagnosed with PTO were eligible for our study. The local Institutional Review Board (Fundação Faculdade Regional de Medicina S J Rio Preto) approved the study, under the protocol number: 234.654.

### Description of experiment, treatment, or surgery

Osteomyelitis was defined based upon the Center for Disease Control and Prevention (CDC)/National Healthcare Safety Network (NHSN) guidelines [15]. We considered patients in remission of infection when there was absence of clinical, laboratory, or radiological signs of infection evaluated during the last medical visit (minimum of one year of follow-up), and in cases in which there was no need for reoperation or administration of an extra course of antibiotic therapy for the same site of infection following the end of therapy [11, 15]. Treatment failure or recurrent infection was defined as infection at the same site that had been previously controlled and required reoperation and/or a second complete course of parenteral antibiotic therapy [13, 15–18]. For the purpose of study analysis, we included only the first episode of recurrence and subsequent episodes were further excluded.

### Variables, outcome measures, data sources, and bias

In order to identify potential risk factors associated with failure of treatment of PTO, several variables (patient comorbidities, injury, microbiological findings, and surgery associated variables) were assessed by reviewing medical, intra-operative, and microbiological records. Demographics, comorbidities, smoking, alcohol consumption, diabetes and American Society of Anesthesiologists (ASA) classification were also analyzed. Injury-associated variables assessed included time elapsed from admission to the first dose of antibiotic and to surgery, anatomical site of fracture, mechanism of trauma such as low-energy injury vs high-energy (based on the energy of the mechanism), and Gustilo type. Surgery-related factors analyzed were type of surgical procedure (open reduction and internal fixation or two-stage fixation with temporary external fixator), duration of surgery, and the need for blood transfusion. In addition, we assessed the need to perform supplementary surgical debridement for infected wounds. Specimen collection for microbiology and pathology was performed in the OR with a minimum of three tissue samples from infected bone and soft tissues at the time of surgical debridement.

### Statistical analysis, study size

For statistical analysis, the follow-up was defined as the time interval between the date of the first medical visit and date of remission or failure of treatment of PTO, considering at least one-year of follow-up. The association between qualitative variables was made using the chi-square test and Fisher’s exact test, and between quantitative variables bivariate logistic regression was utilized. The risk estimates were calculated on the variables associated with risk factors for failure and reported as a hazard ratio with respect to 95% confidence interval (CI). Multiple logistic regression model by selecting the variables of bivariate analysis tests was applied when there were significance levels lower than 0.20 (*p <* 0.20) and remained in the final models only significant variables lower than 0.5 (*p <* 0.05). The Kaplan-Meier method was used to calculate disease-free survival, and log rank test to evaluate the equality of survival distributions across different strata. Disease-free survival was also evaluated using univariate and multivariate Cox regression analysis. The difference was considered statistically significant if the *p-*value was less than to 0.05. All data were analyzed using SPSS version 23 (IBM-SPSS Inc., Chicago, IL, USA).

## Results

**Fig. 1:**
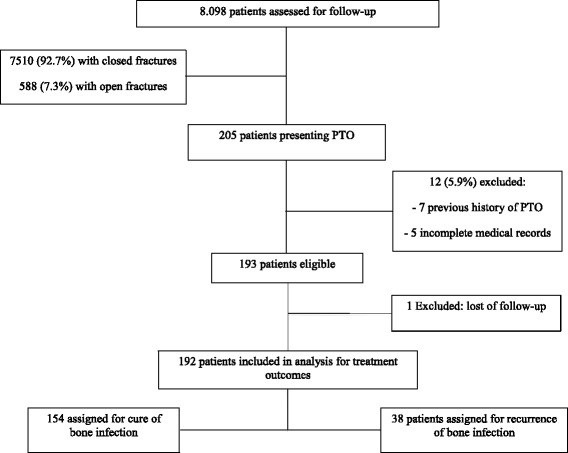
Flow diagram indicating patient enrollment and follow-up throughout the trial

During the study period, one hundred and ninety-three patients with PTO were included, although one patient was excluded from further analysis due to loss of follow-up. Therefore, 192 patients were included for the outcome analysis, of which 154 (80.2%) presented with remission of bone infection at final follow up, and 38 (19.8%) were diagnosed with recurrent infection (Fig. 1). Demographic, clinical, and injury characteristics of the study population are summarized in Table 1. Sixty-six (34.2%) subjects needed intraoperative blood transfusion. Nearly two-thirds (62%) of our PTO cases (120 patients) were associated with closed fractures, whereas open fractures occurred in 73 patients (38%). Although, in our cohort of PTO patients the rates of closed and open fractures were 1.6% (120∕7510) and 12% (73∕588), respectively. Patient, injury, surgical and microbiological related factors that were investigated for possible association with treatment failure of PTO in the univariate analysis are shown in Table 2. Of note, patients with higher risk for treatment failure were female (failure in 50% *vs.* remission in 26.6%, *p* = 0.005), with older age (median age of 70 years *vs.* 40.5 years, *p <* 0.001), had undergone additional debridement procedures (60.5% *vs*. 35%, *p* = 0.004), and needed intraoperative blood transfusions (60.5% *vs*. 28%, *p <*0.001) presented higher risk for recurrence of infection after PTO, in the univariate analysis. Additionally, infections caused by *Escherichia coli*, *Pseudomonas aeruginosa* and *Acinetobacter baumannii* also presented higher risk for treatment failure, based on our univariate analysis.

**Table 1:** Demographic and injury characteristics of 193 patients associated with treatment remission and failure of PTO^a^

Demographic data	*N =* 193 (%)
Age (mean [range]) (years)	50 (16–88)
Male sex (no. [%])	133 (68.9)
Comorbidities (no. [%])	
Smoking	59 (30.5)
Intraoperative hyperglycemia	58 (30.0)
Occupation (no. [%])	
Farmer	26 (13.5)
Driver (car, motorcycle, truck)	24 (12.5)
Construction/Machine Operation	44 (22.8)
Business	40 (20.7)
Household activities	59 (30.5)
Types of injury (no. [%])	
Motor vehicle accident	110 (57.0)
Fall from height (<1m)	57 (29.5)
Football related injury	3 (1.5)
Other falls	18 (9.6)
Other injuries	8 (4.0)
Penetrating injury by a wooden foreign body	3 (1.5)
High-energy injury	133 (68.9)
Gustilo & Anderson Classification for open fractures 73 (37.8)	
Type-I (no. [%])	14 (19.2)
Type-II (no. [%])	23 (31.5)
Type-III (no. [%])	36 (49.3)
Closed fracture	120 (62.2)
More of one surgical debridement	77 (39.9)
Polytrauma	46 (23.8)
American Society of Anesthesiologists score (no. [%])	
ASA^b^ I – II (1 or 2)	166 (90.7)
ASA^b^ III – IV (>2)	17 (9.3)
Intraoperative blood transfusion	66 (34.2)
Duration of surgery (media [range]) (hours)	2.61 (1–8)
Infected Fracture Location (no. [%])	
Upper limbs	44 (22.8)
Lower limbs	154 (79.2)
Collarbone	7 (3.6)
Humerus	12 (6.2)
Radius	13 (6.7)
Ulna	16 (8.2)
Hands	3 (1.5)
Femur	59 (30.5)
Knee	9 (4.6)
Tibia	56 (29)
Fibula	11 (5.7)
Foot	19 (9.8)

PTO^a^: posttraumatic osteomyelitis

ASA^b^: American Society of Anesthesiologists

**Table 2:** Univariate analysis of risk factors associated with treatment failure of PTO^a^

Characteristics	Failure No. (%) (*N =* 38)	Remission No. (%) (*N =* 154)	*P* value^c^
Demographic data			
Age (median [range]) (yr)	70 (16 – 88)	40.5 (11 – 86)	<0.001
≤40 years	9 (23.6)	77 (50)	
41 – 60 years	6 (15.8)	55 (35.7)	
61 – 80 years	14 (37)	20 (13)	
≥ 80 years	9 (23.6)	2 (1.3)	
Male sex (no. [%])	19 (50)	113 (73.4)	0.005
Household activities (no. [%])	20 (52.6)	39 (25.3)	0.001
Comorbidities (no. [%])			
Smoking	11 (29.0)	50 (32.5)	0.676
Fall from height (no. [%])	23 (60.5)	34 (22.0)	<0.001
Grade-III open fracture (no. [%])	6 (15.8)	30 (19.5)	0.602
Admission to surgery t >3h (no. [%])	36 (94.7)	139 (90.2)	0.532
>1 surgery debridement (no. [%])	23 (60.5)	54 (35.0)	0.004
Need for blood transfusion (no. [%])	23 (60.5)	43 (28.0)	<0.001
Lower limb fractures (no. [%])	37 (97.3)	116 (75.3)	0.002
Bipolar hip prosthesis (no. [%])	8 (21.0)	5 (3.2)	<0.001
ASA score			
ASA^b^ I - II (no. [%])	30 (79.0)	145 (94.1)	0.007
ASA III - IV (no. [%])	9 (12.3)	8 (7.3)	
Chronic osteomyelitis (no. [%])	16 (42.1)	84 (54.5)	0.169
*Staphylococcus aureus* culture (no. [%])	25 (13%)	79 (41.1%)	0.010
*Streptococcus* sp. culture (no. [%])	4 (10.5)	4 (2.6)	0.051
*Enterococcus* sp. culture (no. [%])	8 (21.1)	14 (9.1)	0.048
*Escherichia coli* culture (no. [%])	6 (15.8)	8 (5.2)	0.036
*Pseudomonas aeruginosa* culture (no. [%])	17 (44.7)	14 (9.1)	<0.001
*Acinetobacter baumannii* culture (no. [%])	10 (26.3)	13 (8.4)	0.005

PTO^a^: posttraumatic osteomyelitis

ASA^b^: American Society of Anesthesiologists

^c^Patient characteristics were summarized as frequencies and percentages or median and compared using the Pearson Chi-Square test or Fisher’s exact test as appropriate to nominal variables and Mann–Whitney Test or *T*-Test as appropriate to continuous variables. All tests were two sided, and *P* values of <0.05 were considered statistically significant

**Fig. 2:**
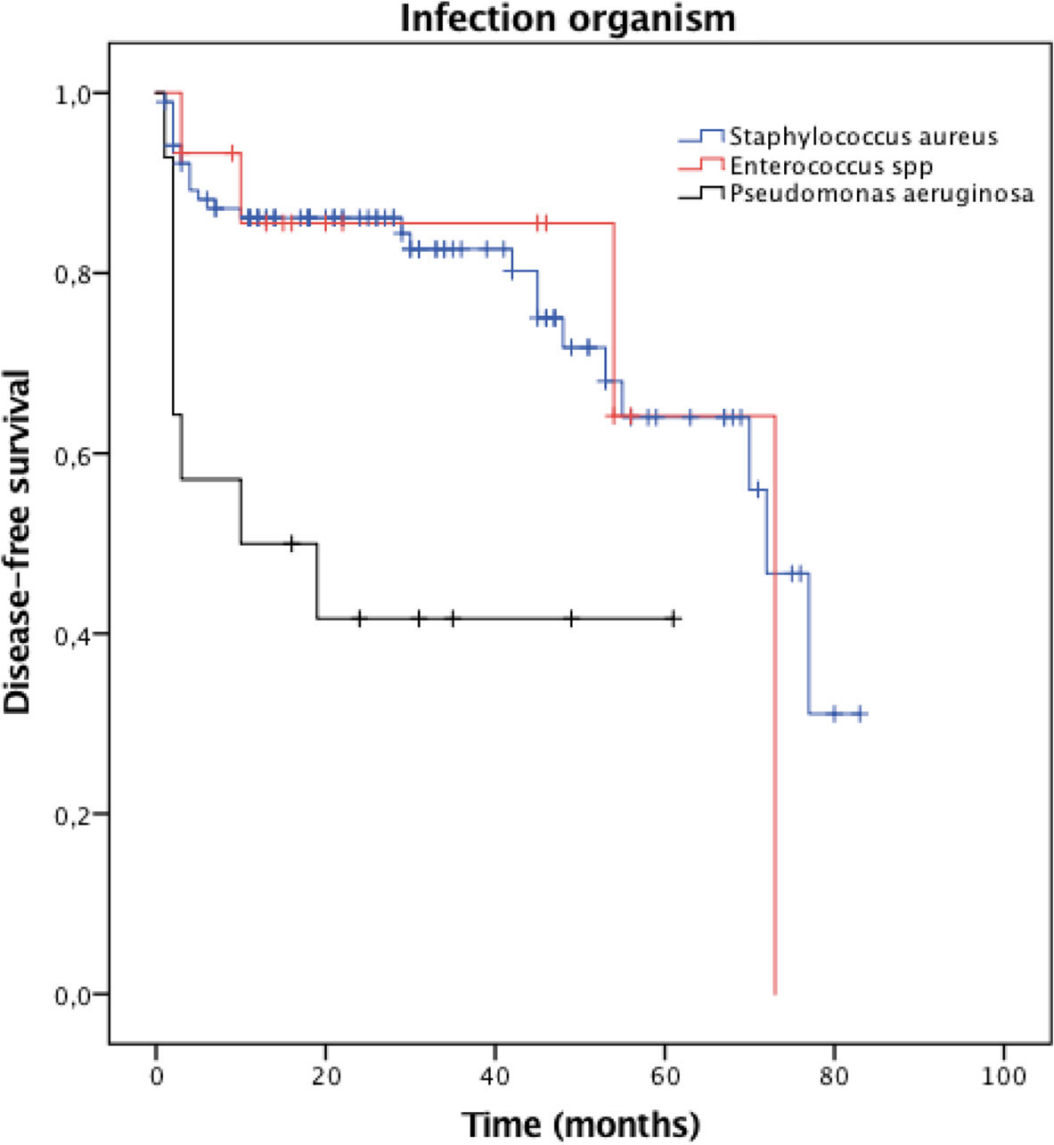
Kaplan–Meier estimates of the cumulative risk of failure according to the treatment assessed at 100 months’ follow-up. The log rank test compared two survival curves across multiple time points. Patients who had PTO caused by *P. aeruginosa* had lower disease-free survival than by *S. aureus* and *Enterococcus* sp. (*p* = 0.002)

**Fig. 3:**
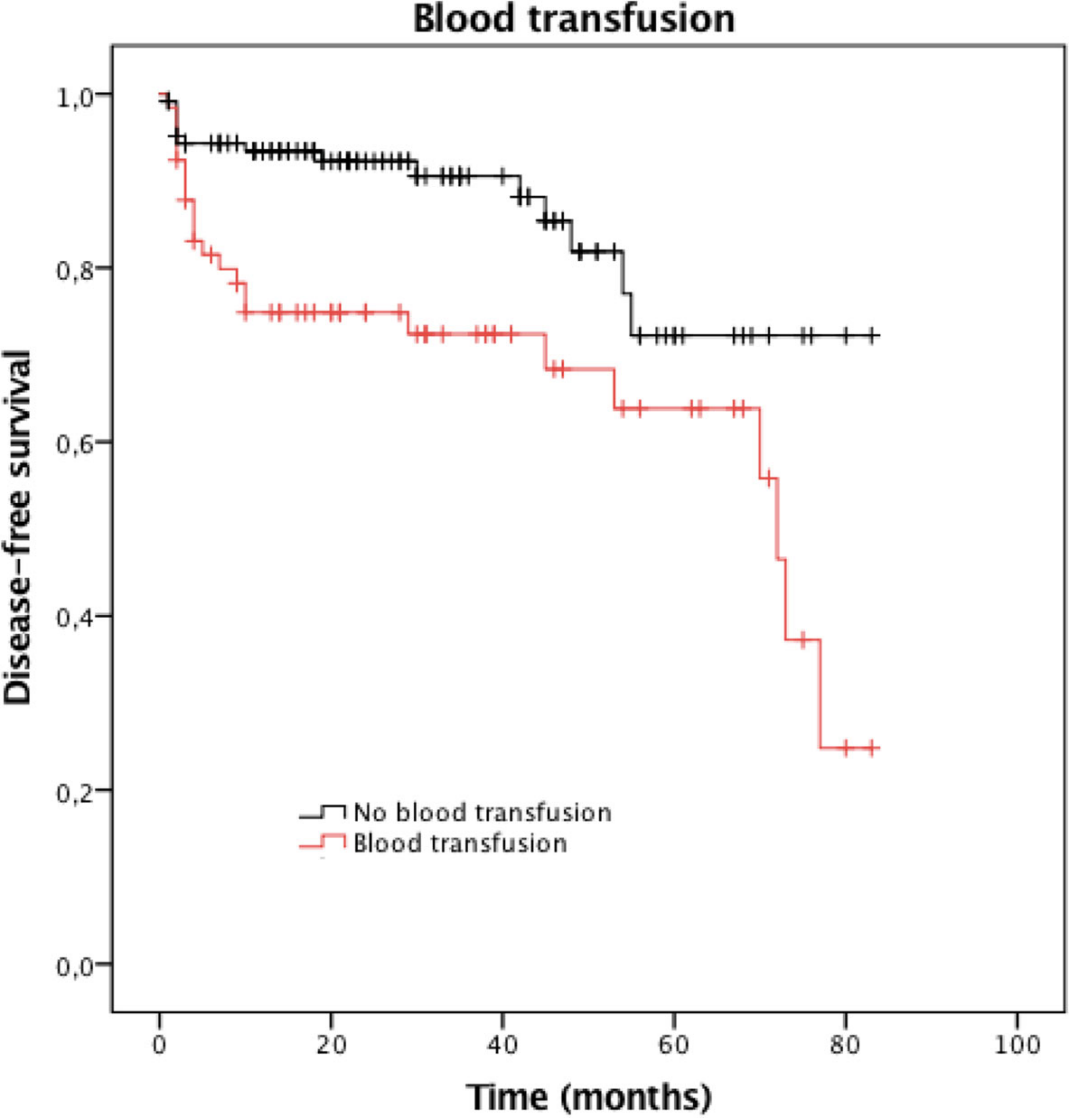
Kaplan–Meier estimates of the cumulative risk of failure according to the treatment assessed at 100 months’ follow-up. The log rank test compared two survival curves across multiple time points. Patients who need of blood transfusion had lower disease-free survival than those who did not need transfusion (*p* = 0.003)

Variables showing statistical significance and presenting clinical importance in univariate analysis were added to the multivariate Cox proportional hazard model (Table 3). Factors that remained independently associated to recurrent infection in the multivariable analysis were elderly patients, especially those with age ranging from 61 to 80 years [hazard ratio (HR) = 6.086, 95% confidence interval (CI) = 2.459;15.061, *p <*0.001], and above 80 years [HR = 9.975 (95% CI = 3.591;27.714), *p <*0.001]; need for intraoperative blood transfusion [HR = 2.239 (95% CI = 1.138;4.406), *p =* 0.020]; and positive bone and/or soft tissue culture for *P. aeruginosa* [HR = 2.700 (95% CI = 1.370;5.319), *p =* 0.004]. We have found a strong correlation between *P. aeruginosa* and treatment failure of PTO. When *P. aeruginosa* was the recovered pathogen, disease-free survival was much lower than that of *S. aureus* and *Enterococcus* sp. (28 months *vs.* 60 months *vs.* 59 months, respectively, *p =* 0.002), on the Kaplan-Meier survival curve of all 192 patients with PTO (Fig. 2). Regarding the need for intraoperative blood transfusion, disease-free survival was of 1681 days for those who received red blood cell transfusion *vs.* 2136 days for those who did not received transfusion (*p =* 0.003) (Fig. 3). The frequencies of microorganisms isolated from bone and soft tissue cultures among patients presenting with PTO are shown in Table 4. Of the 300 positive cultures, *Staphylococcus aureus* was isolated in 104 (34.7%) cases, of which 37 (12.3%) and 67 (22.3%) were methicillin-resistant *S. aureus* (MRSA) and methicillin-sensitive *S. aureus* (MSSA), respectively.

**Table 3:** Multivariate Cox proportional hazard model of risk factors associated with treatment failure of PTO^d^

Variables	HR^a^ (CI 95%)	*P* Value^c^
Age^b^		
61 – 80 years	6.086 (2.459; 15.061)	<0.001
> 80 years	9.975 (3.591; 27.714)	<0.001
Need for blood transfusion	2.239 (1.138; 4.406)	0.020
*Pseudomonas aeruginosa* culture	2.700 (1.370; 5.319)	0.004

^a^Hazard Ratio

^b^Less than or equal 40 years as reference

^c^All tests were two sided, and *P* values of <0.05 were considered statistically significant

PTO^d^: posttraumatic osteomyelitis

**Table 4:** Frequency of microorganisms yielded from cultures

Microrganisms	N (%)
*Staphylococcus aureus*	104 (34.6)
MRSA^a^	37 (12.3)
MSSA^b^	67 (22.3)
*Pseudomonas aeruginosa*	31 (10.3)
*Acinetobacter baumannii*	23 (7.6)
*Enterococcus* sp.	22 (7.3)
*Citrobacter* sp.	21 (7.0)
*Klebsiella pneumoniae*	20 (6.6)
CNS^c^	19 (6.3)
*Escherichia coli*	14 (4.6)
*Proteus* sp	14 (4.6)
*Candida tropicalis*	2 (0.6)
Others^d^	30 (10.0)

^a^MRSA-Methicillin-resistant *Staphylococcus aureus*

^b^MSSA- Methicillin-sensitive *Staphylococcus aureus*

^c^CNS-Coagulase-negative Staphylococci

^d^Others - *Alcaligenes* sp.*, Enterobacter* sp., *Morganella morganii, Providencia* sp.*, Serratia* sp., *Stenotrophomonas maltophilia, Streptococcus agalactie*, *Streptococcus viridans*

## Discussion

Depending upon the severity of the injury and the type of host, PTO may reach rates as high as 50%, and failure to control the infection through aggressive surgical debridement and culture-based antibiotic therapy may be limb-threatening [6, 12, 18–25]. Nevertheless, risk factors for recurrent infection following surgical treatment of PTO have received little attention in the medical literature. The present study reported the outcome of 192 patients that underwent surgical and antibiotic treatment for osteomyelitis. Of these patients, 38 (19.8%) experienced recurrence of infection. We therefore focused on identifying significant predisposing factors for recurrence of osteomyelitis following treatment of PTO. The results identified that elderly patients, intra-operative blood transfusion, and infections caused by *Pseudomonas aeruginosa* were independently associated with a substantially increased risk of treatment failure.

Considerable amount of data has been published regarding the incidence of osteomyelitis following open and closed fractures, with rates ranging from 1% up to 50% [2, 19]. In contrast, data describing the success of PTO management is surprisingly scarce. In this case-series, 80.2% of patients were in remission after at least one year of follow up. A remission rate varying from 69.4% and 82.1% were observed in few retrospective studies of posttraumatic and postoperative osteomyelitis [13, 14, 23]. On the other hand, better remission rate (92%) was observed in an osteosynthesis-associated infection Swiss single-center case–control study [11]. Explaining these discrepancies may be a complex task, but we hypothesize that the variables presented our study, such as, patient characteristics, proportion of high-energy trauma-associated fractures (68.9%), and surgical technique may have played a role. In fact, contrary to the Swiss study population, our study population experienced high-energy trauma fractures due to road traffic accidents ultimately requiring more than one surgical debridement [11].

The major finding of this study is that independent factors for treatment failure following posttraumatic osteomyelitis are essentially non-modifiable. Elderly patients with an age between 61 and 80 years face a six-fold greater risk to recur after PTO management. Patients above 80 years of age have an almost ten-fold risk for treatment failure. In patients receiving blood transfusion, there is a two-fold higher risk of recurrent infection. A 2.7-fold higher risk of treatment failure was noted when the infectious organism was found to be *P. aeruginosa.* A strong association between older age and osteomyelitis has been shown in previous studies to be multifactorial, including immunocompromised features of aging, higher frequency of comorbidities (diabetes and peripheral vascular diseases) [12, 13, 26]. Large blood loss at the time of fracture or during and after surgery causing severe and symptomatic anemia is the main clinical indication for red blood cell transfusion [27]. In addition, transfusion of allogenic blood units may reflect the severity of the injury, producing extensive soft tissue damage and consequently serious bleeding. The literature has shown that intra-operative blood transfusion is not without side effects. Although controversial, previous studies have associated allogenic blood transfusion with periprosthetic joint infection [28, 29]. Conversely, a Cochrane systematic review was unable to identify a strong association between blood transfusion and wound infection following hip fracture trauma [30]. Even though intraoperative blood transfusion is independently associated with recurrent PTO in the present study, this result should be interpreted cautiously. One may speculate that patients submitted to more complex surgeries with prolonged operative time may be more likely to have received blood transfusion. Nevertheless, in the present study, significantly lower infection-free survival rate was observed among patients who had received blood transfusion.

Regardless of the fact that *Staphylococcus aureus* (MSSA and MRSA) comprises by far the most common infecting organism isolated on cultures from PTO, the Gram-negative rod *Pseudomonas aeruginosa* is likely to be identified on soft tissue and bone cultures [3, 4, 7–9, 19]. Contamination by a myriad of pathogenic Gram-negative bacteria is not only found in open fractures, but in recent studies involving trauma centers of developing countries, *P. aeruginosa* has been increasingly identified as the causative organism in secondary hospital-acquired osteomyelitis and implant-associated infections [4, 6, 8, 31, 32]. In this study, *P. aeruginosa* accounted for the second most common isolated bacteria on patients with PTO and was strongly associated with recurrent infections in the multivariate analysis. Furthermore, the disease-free survival was much lower among patients presenting *P. aeruginosa* PTO than that of *S. aureus* and *Enterococcus* sp. on the Kaplan-Meier curve (28 months *vs.* 60 months *vs.* 59 months, respectively). To our knowledge, few studies addressing musculoskeletal infection were able to prove an independent association between positive cultures with *Pseudomonas* and a worse outcome (13). In addition, the majority of data from these previous studies assessed patients with prosthetic joint infections rather than posttraumatic osteomyelitis [15, 23, 28, 33–36]. In our study, the prognostic implication of *P. aeruginosa* on the recurrence of PTO should not a surprise, as this major human opportunistic pathogen has shown a range mechanisms of adaptation, survival (biofilm formation), expression of important virulent factors and yet, expressing resistance to almost all classes to antibiotics. By understanding that the causative organism may provide prognostic clues, orthopedic surgeons and infectious disease specialists should work on preventive measures targeted at Gram-negative infections and have a more aggressive approach to seek a microbial diagnosis upon clinical suspicion of PTO.

This single-center study has potential limitations including its retrospective design along with the small number of patients with recurrences (38/192). This may affect the performance of the multivariable-adjusted analysis in previously described associations with recurrent PTO, which include high-energy open fractures [14], presence of osteosynthesis on the site of infection [11] and inappropriate use of antibiotics [13, 15]. However, disease-free survival analysis over time was also performed in this study using univariate and multivariate Cox regression analysis. The study was carried out at a single public institution in a medium-sized city in a developing country offering specialized orthopedic care for the local population, from which results may not be applicable to centers in differing locations. However, a large single-center database of orthopedic trauma surgeries (roughly 8,000) was used for this analysis. Recurrent PTO may have been misdiagnosed over time due to the absence of typical signs and symptoms of inflammation especially for osteosynthesis-associated infections [16, 19]. On the other hand, follow-up of patients with PTO following hospital discharge and treatment completion is usually performed by the same team of experienced orthopedics and infectious diseases specialists in an outpatient setting within the same institution, and therefore, this selection bias is considered well controlled. In this study, a minimal follow-up time was one year; although a median follow-up time of 1395 days was observed further limiting the selection of bias.

## Conclusions

Despite the highlighted limitations, this single-center retrospective study revealed a high rate of recurrence following PTO in a specific subset of patients and clinical scenarios. Higher rates of recurrence in patients who develop PTO were seen in elderly patients, patients requiring intra-operative blood transfusions, and infections caused by *P. aeruginosa.* Future prospective multicenter randomized studies are needed to corroborate these results.

## Abbreviations

ASA: American society of anesthesiologists
CDC/NHSN: Center for diseases control and prevention/national health care safety network
CI: Confidence interval
CNS: Coagulase-negative Staphylococci
*COM*: Chronic osteomyelitis
HR: Hazard ratio
MRSA: Methicillin-resistant *Staphylococcus aureus*
*MSSA*: Methicillin-sensitive *S. aureus*
PTO: Posttraumatic osteomyelitis
